# Diagnostic accuracy of existing methods for identifying diabetic foot ulcers from inpatient and outpatient datasets

**DOI:** 10.1186/1757-1146-3-27

**Published:** 2010-11-24

**Authors:** Min-Woong Sohn, Elly Budiman-Mak, Rodney M Stuck, Farah Siddiqui, Todd A Lee

**Affiliations:** 1 Center for Management of Complex Chronic Care, Edward Hines, Jr. VA Hospital, Hines, IL, USA; 2 Institute for Healthcare Studies, Feinberg School of Medicine, Northwestern University, Chicago, IL, USA; 3 Department of Medicine, Loyola University Stritch School of Medicine, Maywood, IL, USA; 4 Surgical Service, Edward Hines, Jr. VA Hospital, Hines, IL, USA; 5 Department of Orthopaedic Surgery, Loyola University Stritch School of Medicine, Maywood, IL, USA; 6 Department of Plastic Surgery, Georgetown University Hospital, Washington, DC, USA; 7 Center for Pharmacoeconomic Research, Departments of Pharmacy Practice and Pharmacy Administration, College of Pharmacy, University of Illinois at Chicago, Chicago, IL, USA

## Abstract

**Background:**

As the number of persons with diabetes is projected to double in the next 25 years in the US, an accurate method of identifying diabetic foot ulcers in population-based data sources are ever more important for disease surveillance and public health purposes. The objectives of this study are to evaluate the accuracy of existing methods and to propose a new method.

**Methods:**

Four existing methods were used to identify all patients diagnosed with a foot ulcer in a Department of Veterans Affairs (VA) hospital from the inpatient and outpatient datasets for 2003. Their electronic medical records were reviewed to verify whether the medical records positively indicate presence of a diabetic foot ulcer in diagnoses, medical assessments, or consults. For each method, five measures of accuracy and agreement were evaluated using data from medical records as the gold standard.

**Results:**

Our medical record reviews show that all methods had sensitivity > 92% but their specificity varied substantially between 74% and 91%. A method used in Harrington et al. (2004) was the most accurate with 94% sensitivity and 91% specificity and produced an annual prevalence of 3.3% among VA users with diabetes nationwide. A new and simpler method consisting of two codes (707.1× and 707.9) shows an equally good accuracy with 93% sensitivity and 91% specificity and 3.1% prevalence.

**Conclusions:**

Our results indicate that the Harrington and New methods are highly comparable and accurate. We recommend the Harrington method for its accuracy and the New method for its simplicity and comparable accuracy.

## Background

With the rapid spread of electronic medical records, there is a growing need for accurately identifying health conditions through electronic medical records in order to establish population-based rates for disease surveillance purposes and to cost-effectively identify patients for targeted interventions and research studies. Diabetic foot ulcers (DFUs) are significant public health concerns due to high economic burden [1-4], negative impact on quality of life [5,6], and their association with increased risk of amputation [7,8] and premature death [9,10]. However, their national estimates of incidence or prevalence rates are not currently available, possibly due to the lack of a reliable method to identify this condition in administrative health data. We only know that a lifetime risk of foot ulceration for a diabetic patient may be as high as 25% [11] and that annual incidence and prevalence rates may be as high as 4% and 10% in selected populations [12,13].

Four different methods [1-3,14] have been used in previous observational studies. They differed considerably from one another in complexity and sophistication; they were designed for different purposes and were used with different databases. In a study of costs and duration of treatment for foot ulcer patients, Holzer and colleagues [2] identified DFU patients from inpatient and outpatient claims data. Any patient with one or more claims containing a foot ulcer-related diagnosis or procedure in any fields was identified as having the DFU diagnosis.

In a descriptive study of inpatient care for patients with lower-extremity complications of diabetes, Mayfield et al. [14] reported that over 18,000 hospitalizations for lower-extremity complications occurred in 1998. They identified foot ulcers using a method consisting of diagnostic codes only. Venous stasis ulcers and decubitus ulcers were excluded but surgical complications from a stump infection, an orthopaedic procedure, or a prior vascular graft in the foot were identified as a DFU.

Ramsey et al. [3,15] used the simplest method, involving only one diagnostic code (ICD-9-CM 707.1×, "Ulcer of lower limbs, except decubitus"), in a study of incidence rates and treatment costs of foot ulcers among individuals enrolled in a HMO. In a validation study, this method was shown to have 74% sensitivity and 94% specificity compared to medical records [15].

Finally, the method used in Harrington et al. [1] was based on diagnostic codes used in the Holzer method [2] discussed above. The Harrington method, however, further required that some conditions such as osteomyelitis or gangrene should be confirmed with foot-specific procedures, because ICD-9-CM codes for these conditions did not identify body parts where they occurred. In this method, patients were identified as having a DFU if they had ICD-9-CM codes 707.1×, 707.8 ("Chronic ulcer of other specified sites"), or 707.9 ("Chronic ulcer of unspecified sites") in any field in administrative data or if they had any other ulcer-related diagnoses used in the Holzer method that were confirmed by subsequent procedures on the foot. These methods are summarized in Table 1.

**Table 1:** Existing methods of identifying diabetic foot ulcers in administrative data

	ICD-9-CM or CPT-4 codes	Holzer	Mayfield	Harrington
**A. Lower-extremity ulcer diagnosis**				
Ulcer of lower limbs	707.1×	x^1^	x	X
Chronic ulcer of other specified sites	707.8	x		X
Chronic ulcer of unspecified sites	707.9	x	x	X
Carbuncle and furnancle of foot	680.7			Xx
Cellulitis and abscess of toe or foot	681.1, 682.7	x	x	Xx
Cellulitis and abscess of unspecified digit	681.9	x		Xx
Other cellulitis and abscess, leg except foot	682.6		x	
Osteomyelitis^2^	730.06-730.09, 730.16-730.19, 730.26-730.29	x	x	Xx
Gangrene^3^	785.4	x	x	Xx
Surgical complications from a stump infection	768		x	
Surgical complications from amputation	997.6		x	
Complications from a prior vascular graft	440.3, 996.62, 996.7, 996.74, E878.2		x	

**B. Lower-extremity ulcer-related procedures**				
Simple repair of superficial wound	12001-12002, 12004-12007	x		xxx
Debridement^4^	11040-11044, 77.68, 86.22, 86.28	x		xxx
Surgical debridement and drainage of abscess and cavities	20005, 28001-28005	x		
Lower-extremity radiographic techniques	73620-73630, 73650-76660			xxx
Angioscopy, arteriography, angiography	75710, 75716			xxx
Lower-extremity CAT or MRI scanning	73700-73702, 73720-73721			xxx
Incision or excision of foot	28001-28008, 28111-28160			xxx
Unna boot application	29540, 29550, 29580			xxx
Culture and sensitivity testing	87040, 87071-87072, 87075-87076, 87082-87085	x		
Aspiration, incision and drainage of infection or abscess	10060-10061, 10160, 20000, 86.01, 86.04	x		
Foot-sparing surgery	28020-28024, 28060, 28070, 28072, 28086, 28088, 28110-28126, 28140, 28150, 28153, 28160, 77.38, 77.88, 80.18	x		
Late amputation stump complication^5^	997.60-997.62, 997.69			xxx
Amputation, foot^6^	28800-28825, 84.10-84.12	x		xxx
Amputation, ankle/leg	27880-27889, 84.13-84.15	x		xxx
Amputation, knee and above	27590-27598, 84.16-84.17	x		xxx

^1 ^'x' indicates the code(s) were used; 'xx' indicates the codes were used only when corroborated by procedures (identified by 'xxx') on or after the date of diagnosis.

^2 ^Mayfield used 729.4, 730.x, and 731.x for osteomyelitis.

^3 ^Mayfield used 785.4, 040.0, and 440.24 for gangrene.

^4 ^Harrington did not use ICD-9 procedure codes.

^5 ^These are ICD-9 diagnostic codes indicating previous surgical procedures.

^6 ^Holzer did not use 84.10.

The objectives of this study were to compare these four methods for their diagnostic accuracy by evaluating them using medical records as the gold standard and to propose a new and simpler method.

## Methods

### Study cohort and data sources

To evaluate the diagnostic coding accuracy of these methods, we first identified all individuals who used the Department of Veterans Affairs (VA) healthcare services in the fiscal year 2003 (October 1, 2002-September 31, 2003; all years hereafter are fiscal years) from the VA national patient care datasets. These datasets contain all records of acute inpatient or outpatient care provided in the US. Patients were identified as having diabetes if they received at least one prescription for a diabetes medication in the current year or if two or more records with diabetes diagnosis (ICD-9-CM 250.xx) existed for inpatient admissions or outpatient visits over a 24-month period (2002-2003). This method is known to have 93% sensitivity and 98% specificity relative to self reports of diabetes [16].

From the national diabetic cohort (N = 866,881), we identified all patients who used healthcare services exclusively at a tertiary care hospital in 2003. We identified 4,158 diabetic patients from whom we drew a stratified sample consisting of all individuals who had DFUs according to at least one of the four methods and an equal number of individuals who were randomly selected from those who did not. This resulted in a hospital-based sample of 518 individuals, which we will call the "local" sample below.

### Review of medical records

We provided two authors (EB and FS) with a list of 518 individuals that did not have any indication of whether a diagnosis of a foot ulcer was found in administrative data. EB and FS divided the list into half and independently reviewed patients' electronic medical records. Their aim was to determine whether a diabetic foot ulcer was indicated on medical records in 2003. A diabetic foot ulcer was conceptually defined as a full-thickness break of the integument on a diabetic foot. It was indicated if there was any explicit mention of "diabetic foot ulcer" or any qualifying wound or lesion on an ankle or a foot was noted on medical records. When osteomyelitis or gangrene was mentioned alone in 2003, we identified it as a DFU if we could link it to foot ulceration on the same foot and location in 2002. Osteomyelitis due to puncture wounds, gangrene due to arterial occlusion/embolic phenomenon, abrasions, venous stasis ulcers, and decubitus ulcers were excluded from the case definition.

There were 45 cases whose DFU status could not be unambiguously determined by the reviewers. These cases were examined by both EB and FS and a third reviewer (RS). When there were disagreements between EB and FS, we used the opinion of the third reviewer to adjudicate the case. To assess inter-rater reliability, we randomly selected 30 medical records *de novo *from the "local" sample and all three reviewers (EB, FS, RS) independently conducted the reviews. Cronbach's alpha for the inter-rater reliability among three reviewers was 0.93, indicating a high consistency.

### New identification method

In addition to evaluating existing methods, we developed a new, simple method for DFU identification. The New method consisted of two codes 707.1× and 707.9 documented in any position on an inpatient or outpatient encounter. These two codes were common to the Holzer, Mayfield, and Harrington methods and thus the New method will identify a subset of patients also identified by the first three methods.

### Statistical analysis

Foot ulcer indication in medical charts was used as the "gold standard" against which four methods were evaluated for diagnostic accuracy. Sensitivity and specificity were computed for each method. Sensitivity indicates the probability that a foot ulcer indication on medical charts is correctly identified by a method. Specificity indicates the probability that a patient who does not have an indication on medical charts is not identified as having the condition by a method. We additionally computed weighted positive predictive value (PPV) and negative predictive value (NPV) to account for disproportionate sampling in the "local" sample [17]. PPV indicates the proportion of patients a method correctly predicts a foot ulcer indication on medical records and NPV, the proportion a method correctly excludes as not having a foot ulcer indication on medical records. Simple kappa, weighted to adjust for bias due to disproportionate sampling, was computed for each method as a measure of agreement between administrative data and medical charts [18,19]. Sampling weights used for PPV, NPV, and kappa were the inverse of the probability of selection to the local sample.

The study was approved by the Institutional Review Board at the Hines VA Hospital.

## Results

### Prevalence rates of diabetic foot ulcers based on four methods

We identified 866,881 patients who used VA healthcare services in the US in 2003 with a diagnosis of diabetes. They were 68 ± 11 years old, mostly male (98%) and non-Hispanic whites (71%). Sixteen percent were newly diagnosed with diabetes in 2003 and 24% had had diabetes for 6 years or longer.

Annual prevalence rates of diabetic foot ulcers ranged between 2.7% and 3.9% from method to method (Table 2). The Ramsey method identified the smallest and the Mayfield method the largest number of DFU patients, with the latter identifying 41% more than the former. The other two methods produced prevalence rates of 3.6% (Holzer) and 3.3% (Harrington).

**Table 2:** Diabetic foot ulcer prevalence according to five methods (N = 866,881)

Method	Cases	Prevalence	Agreement among methods*				
			
			Holzer	Mayfield	Ramsey	Harrington	New
Holzer	31,516	3.64%	-	90.1%	75.3%	89.7%	85.0%

Mayfield	33,533	3.87%	84.7%	-	70.7%	82.0%	79.9%

Ramsey	23,721	2.74%	100.0%	100.0%	-	100.0%	100.0%

Harrington	28,300	3.26%	99.9%	97.2%	83.8%	-	94.7%

New	26,801	3.09%	100.0%	100.0%	88.5%	100.0%	-

* Indicates percent patients identified by the method on the row as having a diabetic foot ulcer is also identified as having an ulcer according to the method on the column. For example, Holzer method identified 84.7% of all patients identified by Mayfield method as having an ulcer.

A comparison among methods shown in Table 2 suggests that Holzer and Mayfield methods identified essentially all patients who were also identified by the other two methods. All other methods captured 100% of those who were identified by the Ramsey method, indicating that the Ramsey method was the least common denominator of all methods.

### Comparison of accuracy

The chart reviews identified 156 individuals in the local sample as having a foot ulcer indication. Table 3 shows accuracy and agreement measures for the four methods. All methods had high sensitivity and NPV. Sensitivity ranged between 92.3% for the Ramsey method to 97.4% for the Mayfield method. NPVs for all methods were greater than 98%. On the other hand, specificity and PPVs varied widely. The Mayfield method had the lowest specificity (73.8%) and PPV (61.5%) due to a large number of false positives (95 patients), followed by the Holzer method with 59 false positives. The other two methods had specificity > 90% and PPV > 80%. Kappa ranged between 0.64 (Mayfield) and 0.73 (Ramsey and Harrington).

**Table 3:** Comparison of methods for diagnostic accuracy of diabetic foot ulcers (N = 518)

Method		Chart review*		Accuracy and agreement measures (95% CI)^†^				
		
		Yes	No	Sensitivity	Specificity	PPV	NPV	Kappa
Holzer	Yes	148	59	94.9	83.7	71.5	98.4	0.69
	No	8	303	(90.1-97.8)	(79.5-87.4)	(64.8-77.5)	(97.9-98.7)	(0.66-0.72)

Mayfield	Yes	152	95	97.4	73.8	61.5	98.5	0.64
	No	4	267	(93.6-99.3)	(68.9-78.2)	(55.2-67.6)	(98.0-98.8)	(0.61-0.67)

Ramsey	Yes	144	31	92.3	91.4	82.3	98.3	0.73
	No	12	331	(86.9-96.0)	(88.1-94.1)	(75.8-87.6)	(97.8-98.7)	(0.70-0.76)

Harrington	Yes	147	36	94.2	90.1	80.3	98.4	0.73
	No	9	326	(89.3-97.3)	(86.5-92.9)	(73.8-85.8)	(97.9-98.7)	(0.70-0.76)

New	Yes	145	33	92.9	90.9	81.5	98.3	0.73
	No	11	329	(87.7-96.4)	(87.4-93.6)	(75.0-86.9)	(97.9-98.7)	(0.70-0.76)

* Chart reviews identified whether there was any indication of a diabetic foot ulcer in the electronic medical records during October 1, 2002-September 30, 2003.

^† ^PPV refers to positive predictive values and NPV, negative predictive values. PPV, NPV, and kappa coefficients were weighted.

The Ramsey method was similar in all measures to the Harrington method, but the former can capture only 83% of DFU patients identified by the latter in the national diabetic population as shown in Table 1. In contrast, the Ramsey method produced the smallest number incorrectly classified (43 false positives plus true negatives, 8.3% of the local sample), followed by the Harrington method with 45 (8.7%). The other two methods fared worse with 67 for the Holzer (12.9%) and 99 (19.1%) for the Mayfield method.

We found that a fifth method ("New" in Tables 2 and 3) that consisted of two codes 707.1× and 707.9 performed as well as the Harrington method with 92.9% sensitivity and 90.9% specificity and 44 (8.5%) incorrectly classified. Kappa for the New method was 0.73, indicating substantial agreement with medical records [20].

## Discussion

Our objective in this study was to evaluate diagnostic coding accuracy of four existing methods compared to medical records. We showed that the five methods we examined in this study performed very well in sensitivity. Holzer and Mayfield methods identified a large number of false positives with a resulting low specificity and positive predictive values. The last three methods (Ramsey, Harrington, and New) had sensitivity > 92% for coding accuracy and were similar in specificity (90.1-91.4), even though the number of diagnostic and procedure codes involved varied considerably. We also showed that the DFU prevalence based on five methods varied considerably. The Mayfield method identified 41% more cases than the Ramsey method, suggesting that the choice of a method can substantially influence prevalence estimates.

As far as we know, the Ramsey method was the only one that was previously evaluated for accuracy. Compared with medical records for patients enrolled in a commercial healthcare plan, this method had 74% sensitivity and 94% specificity [15]. A study by Harwell et al. [21] evaluated an algorithm for "foot complications" that included DFUs, Charcot arthropathy, and lower-extremity revascularization or bypass procedures. Their algorithm was based on the Harrington method (for identifying DFUs that comprise the large majority of foot complications) with additional codes for Charcot arthropathy and lower-extremity vascular procedures. This algorithm had excellent accuracy (99% sensitivity and 93% specificity) in identifying foot complications from inpatient administrative records. These results are consistent with ours on the Harrington method, even though sensitivity and specificity are much higher in the Harwell et al. study than in ours. The difference may be attributed to the fact that the results from the Harwell et al. study were obtained from inpatient administrative records and ours from both inpatient and outpatient records, and to the fact that their case definition is much broader ("foot complications") than ours (DFUs).

This study has limitations. The measures of agreement for different methods in this study may not be generalizable to non-VA databases to the extent that the practices for coding foot ulcers are different from system to system. In principle, the VA uses coding guidelines that are also used in the rest of the medical community, namely, the Official Guidelines for Coding and Reporting approved by the American Hospital Association, the American Health Information Management Association, the Centers for Medicare and Medicaid Services, and the National Center for Health Statistics [22]. Variation in adherence to these guidelines, coding intensity, and data quality among providers need to be considered when applying the results of this study to non-VA data such as Medicare claims. Further research is also needed to confirm whether our findings based on the VA data can be applied to the non-VA data.

Another limitation is that the disease coding in the administrative data were not matched with medical charts kept on the same date. It was not practicable for us to match every eligible code used in Harrington or Holzer methods with medical charts for the same date. Establishing the accuracy of diagnostic coding for each administrative health record is important for determining, for example, the first date of diagnosis or whether a disease existed before or after the onset of another disease. In a supplemental analysis, we assessed the accuracy at the code-day level by randomly selecting 30 patients with encounters coded with 707.1× or 707.9 in the local sample and matched their encounters with medical charts for the same date. We found that 29 (97%) were corroborated by medical charts, suggesting an excellent accuracy of the New method at the code-day level in the VA data.

## Conclusions

Our chart reviews show that administrative data can be used to identify persons with DFU with considerably higher accuracy than previously believed. The accuracy of DFU identification can be as high as some of the high-risk, high-profile conditions that have received a lot of research and policy attention such as myocardial infarction. Our results indicate that the Harrington and New methods are highly comparable and accurate. We recommend the Harrington method for its accuracy and the New method for its simplicity and comparable accuracy. The Harrington method showed 94% sensitivity and 90% specificity in accuracy in the VA administrative data. According to this method, the annual prevalence of diabetic foot ulcers was 3.3% in the VA diabetic population in 2003.

## List of abbreviations

DFU: Diabetic foot ulcers; NPV: negative predictive value; PPV: positive predictive value; VA: The Department of Veterans Affairs

## Competing interests

The authors declare that they have no competing interests.

## Authors' contributions

MS participated in the conception and design of the study, analyzed the data, and drafted the manuscript; EB obtained funding, participated in the conception and design of the study, conducted medical record reviews, and critically reviewed the manuscript; RS participated in the conception and design of the study, supervised medical record reviews, and critically reviewed the manuscript; FS conducted medical record reviews and critically reviewed the manuscript; TL participated in the design of the study and critically reviewed the manuscript. All authors read and approved the final manuscript.
